# Bacteria in sputum of stable severe asthma and increased airway wall thickness

**DOI:** 10.1186/1465-9921-13-35

**Published:** 2012-04-18

**Authors:** Qingling Zhang, Rowland Illing, Christopher K Hui, Kate Downey, Denis Carr, Martin Stearn, Khalid Alshafi, Andrew Menzies-Gow, Nanshan Zhong, Kian Fan Chung

**Affiliations:** 1 Airways Disease Section, National Heart & Lung Institute, Imperial College, London, UK; 2 State Key Laboratory of Respiratory Diseases, The First Affiliated Hospital, Guangzhou Medical University, Guangzhou, China; 3 Respiratory Biomedical Research Unit, Royal Brompton & Harefield NHS Trust, London, UK; 4 Department of Radiology, Royal Brompton & Harefield NHS Trust, London, UK; 5 Department of Microbiology, Royal Brompton & Harefield NHS Trust, London, UK; 6 Experimental Studies Unit, Imperial College, National Heart and Lung Institute, Dovehouse Street, London SW3 6LY, UK

**Keywords:** Severe asthma, Airway wall thickness, Sputum bacteria

## Abstract

**Background:**

Patients with chronic asthma have thicker intrapulmonary airways measured on high resolution computed tomography (HRCT). We determined whether the presence of lower airway bacteria was associated with increased airway wall thickness.

**Methods:**

In 56 patients with stable severe asthma, sputum specimens obtained either spontaneously or after induction with hypertonic saline were cultured for bacteria and thoracic HRCT scans obtained. Wall thickness (W_T_) and area (W_A_) expressed as a ratio of airway diameter (D) and total area, respectively, were measured at five levels.

**Results:**

Positive bacterial cultures were obtained in 29 patients, with *H. influenzae, P. aeruginosa *and *S. aureus *being the commonest strains. Logistic regression analysis showed that this was associated with the duration of asthma and the exacerbations during the past year. In airways > 2 mm, there was no significant difference in W_A _(67.5 ± 5.4 vs 66.4 ± 5.4) and W_T_/D (21.6 ± 2.7 vs 21.3 ± 2.4) between the culture negative versus positive groups. Similarly, in airways (≤ 2 mm), there were no significant differences in these parameters. The ratio of √wall area to P_i _was negatively correlated with FEV_1_% predicted (p < 0.05).

**Conclusions:**

Bacterial colonization of the lower airways is common in patients with chronic severe asthma and is linked to the duration of asthma and having had exacerbations in the past year, but not with an increase in airway wall thickness.

## Background

The lower airways have until recently been considered to be a sterile environment, and in airway diseases such as bronchiectasis and COPD, the isolation of bacteria such as *Haemophilus influenzae *and *Pseudomonas *species in sputum samples by culture is not an uncommon event [1,2]. While these pathogens are often associated with exacerbations, they are also often present during stable phase of the airways disease indicating chronic colonisation. The isolation of bacterial pathogens in chronic asthma by culture remains understudied. In one report, 27% of asthmatic patients presenting with an exacerbation of asthma had bacteria in sputum with *Streptococcus pneumonia, Streptococcus pyogenes, Staphylococcus aureus, Moraxella catarrhalis *and *Haemophilus influenzae *[3]. This spectrum of bacterial species was also isolated from induced sputum samples in 15% of patients during a stable period of asthma [4]. The more sensitive technique of 16S ribosomal RNA microarray to detect bacterial strains in lower airway epithelial brushings, has revealed an increase in bacterial burden and diversity in patients with mild to moderate asthma compared to non-asthmatic individuals [5,6]. Thus, there may be an increased propensity for asthmatics to carry more bacterial pathogens in their lower airways.

The role of pathogenic bacteria in the lower airways of patients with asthma is unclear. Bacteria through the activation of the innate immune response such as the toll-like receptors may induce the release of inflammatory cytokines such as IL-8 and TNFα that could induce neutrophilic inflammation. Asthma is usually characterised by a chronic inflammatory process that is driven by many factors including Th-2 derived cytokines and airway wall remodeling processes that results in subepithelial fibrosis and an increase in airway smooth muscle mass [7]. Bacterial infections may also contribute to airway wall remodeling through the activation of fibrosis by the release of growth factors such as TGFβ, induced by bacterial lipopolysaccharide, leading to fibroblast activation and release of extracellular matrix proteins [8]. In addition, bacterial products may induce goblet cell hyperplasia and glandular hypertrophy. These changes may be reflected in an increase in airway wall thickness detectable on a high resolution computed tomogram. We therefore hypothesized that the presence of pathogenic bacteria may be associated with an increase in airway wall remodeling that would be reflected in a greater wall thickness. Previous studies using HRCT scans have reported an increase in airway wall thickness in patients with asthma, with the greatest responses seen in those with more severe disease [9-11].

We studied severe asthma patients defined as having persistent symptoms of chronic asthma with frequent exacerbations despite being on maximal treatment medications for their disease [12,13]. We determined the prevalence of pathogenic bacteria that can be cultured from sputum samples and measured airway wall thickness using HRCT scans.

## Methods

### Subjects

Patients with severe asthma were prospectively recruited from the Severe Asthma clinic at the Royal Brompton Hospital, London, over a 6-month period. Asthma was diagnosed on the basis of chronic symptoms and/or of recurrent exacerbations together with previously documented reversible airflow obstruction of > 15% either spontaneously or with treatment. Asthma was considered severe because of persistent symptoms and/or recurrent exacerbations despite the use of high dose inhaled steroid therapy, and often in addition to needing regular oral steroid therapy [13]. All patients had been attending the clinic for at least 6 months and had undergone a Severe Asthma Protocol for confirmation of the diagnosis of severe asthma [14]. Patients with an exacerbation of asthma within the last 4 weeks or with a respiratory tract infection requiring antibiotic treatment within 6 weeks were excluded. Patients with bronchiectasis as judged by a high resolution computed tomograms (HRCT) of the lungs were excluded. Patients were included in the study if they provided a sample of sputum either spontaneously or through its induction by inhalation of hypertonic saline. This project was approved by the Royal Brompton and NHLI Ethics Committee and patients provided informed consent (REC reference: 10/H0711/63).

### Lung function and atopic status

FEV_1 _and forced vital capacity (FVC) were measured using a spirometer (Erich Jaeger UK Ltd, Market Harborough, UK) and published predicted values [15]. Single breath diffusing capacity to carbon monoxide was measured and the transfer coefficient factor to carbon monoxide (K_CO_) was calculated according to transfer factor per unit alveolar volume. Lung volumes including residual volume (RV), and total lung capacity (TLC) were measured in a body plethysmograph (Master Lab; Erich Jaeger UK Ltd).

Atopy was defined by the presence of positive skin-prick tests to at least one common aeroallergen including house dust mite, grass and tree pollen, cat dander and dog dander, aspergillus, alternaria and cladasporidium.

### Sputum culture and quantitative bacterial culture

Sputum samples were first obtained by spontaneous production. If this was not possible, the subject then underwent a hypertonic saline challenge in order to induce sputum production [16]. Sputum plugs were separated from saliva using forceps. An aliquot of sputum was selected using a positive displacement pipette and used for quantitative bacteriological culture [17]. Sputum samples were plated and incubated at 37°C in an atmosphere of 5% CO_2 _in air and examined for bacterial growth after 24 and 48 hours. The definition of a significant load of bacteria in the study was a level which resulted in growth to 10 × 10^6 ^colony-forming units (cfu)/ml for any individual pathogenic respiratory bacteria.

In patients in whom there was a positive bacterial growth, one or more repeat sputum samples were subsequently obtained for further bacterial culture.

### High resolution computed tomographic (HRCT) scans

**Figure 1:**
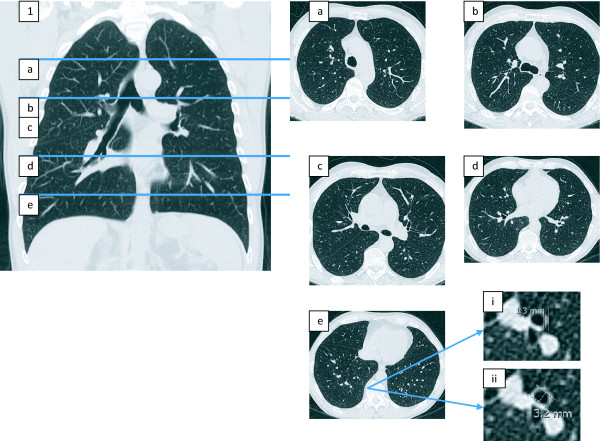
**Coronal reconstruction from a non-contrast high resolution computed tomogram (CT) of the thorax with axial sections marked (a) at the level of the top of the aortic arch, (b) at the carina, (c) 1 cm below the carina, (d) at the level of the midpoint of the right pulmonary vein, and (e) 2 cm above the dome of the right hemi-diaphragm**. The corresponding axial slices are shown (a-e). Representative measurements of airway wall (W) and diameter (D) are shown, taken from slice (e), (i) and (ii) respectively.

HRCT scans were performed on full inspiration on a Somatom 'Sensation' 64-slice CT (Siemens, Erlangen, Germany) using a volume acquisition (120 KVp, 90 mA) with 0.6 mm collimation, a pitch of 1.4 and 1 mm axial reconstructions. The abnormalities of intrapulmonary bronchi (wall thickness and diameter) were obtained at five selected levels according to a previously-published scoring system [18], as shown in Figure 1. The images were viewed at a window level of -500 HU and a window width of 1500 HU. Only the bronchi that were seen as end-on slices were selected.

At each level, one or two bronchial airways were identified which appeared least ovoid and had a short-axis luminal diameter of 1 mm or greater. A minimum of one and maximum of two bronchi were evaluated on each side, at each level of the scan, setting the possible range of bronchi evaluated for each patient as between 10 and 20. If there were more than two candidate bronchi, then the two closest to the mediastinum were chosen. With the bronchus identified, the HRCT images were magnified and measurements made using the electronic calipers built into the picture archiving and communications system (PACS Agfa IMPAX, Germany). The measurements were done by two pulmonary radiologists who had no knowledge of the group which the subject belonged to. A decision was reached by consensus with both radiologists making the assessment together.

We divided the bronchi into 'large' airways with a luminal diameter > 2 mm and 'small' airways with luminal diameter ≤ 2 mm and made airway dimensions on these airways. Wall thickness (W_T_), short-axis luminal diameter (L) and internal perimeter (P_i_) were measured. The total diameter (D) of each airway, total airway area (A_0_), luminal area (A_L_) and wall area (W_A_) were calculated respectively, using the formulae: D = L+2W_T_, A_0 _= π[(2W_T_+L)/2]^2^, A_L _= π(L/2)^2^, W_A _= A_0_-A_L _and Pi = πL. The ratio of airway wall thickness to total diameter (W_T_/D ratio), and the percentage of wall area (W_A_%) defined as (W_A_/A_0_)*100 were used as representative of wall thickness [18,19].

We fitted a linear relationship between P_i _and √W_A _for all airways measured for each subject, and calculated √W_A _at Pi of 10 mm from this relationship. This method condenses all airway thickness measurements into a single summary measure per subject, allowing for comparison of airway wall thickness between subjects while accounting for different airway sizes measured [20].

### Data analysis

Results are given as mean ± SEM. Pearson Chi-square test and Fisher's exact test were used to compare the data between bacteria-negative and bacteria-positive groups where appropriate. Logistic regression analysis was used to assess factors associated with the presence of positive-culture bacteria. Differences were considered significant at p < 0.05.

## Results

### Patient characteristics

Fifty-six patients provided sputum samples that were adequate for analysis of bacterial growth; of these, 16 produced sputum spontaneously and 40 after saline inhalation. Another 9 patients could not provide sputum by either way and were excluded from the study. Twenty-nine of the 56 patients had a significant load of pathogenic bacteria in sputum and 27 did not. A comparison of the demographics and asthma characteristics between these 2 groups is shown in Table 1. The duration of asthma and the total number of asthma exacerbations in the past year were significantly higher in the group with significant bacterial load. There were no significant differences in gender, smoking history (defined as % current and past smoker), obesity (defined as BMI ≥ 30 kg/m^2^), nasal disease, atopy and serum IgE. Lung function was not different between the 2 groups, with a degree of chronic airflow obstruction as expected for severe asthma patients [21]. An equally high proportion of patients in both groups (more than three quarters) were taking oral prednisolone on a daily basis.

**Table 1:** Baseline demographic data

Parameters	Asthma with postive bacteria (n = 29)	Asthma without positive bacteria (n = 27)	P value
Age (years)	54.1 ± 13.4	51.9 ± 123.8	NS

Gender (male)	31.0%	33.3%	NS

BMI, kg/m^2^	29.8 ± 6.1	28.8 ± 5.4	NS

BSA, m^2^	2.0 ± 0.3	1.9 ± 0.3	NS

Obesity %	37.9%	33.3%	NS

Duration of asthma (years)	32.9 ± 17.2	20.5 ± 16.0*	P < 0.05

Smoking % (current and past)	34.5%	48.1%	NS

Nasal diseases%	20.7%	37.0%	NS

Atopy %	41.4%	44.4%	NS

Oral prednisolone %	72.4%	74.0%	NS

Daily prednisolone (mg.day^-1^)	18.5 ± 16.1	19.1 ± 14.3	NS

Inhaled BDP dose-equivalent (mg.day^-1^)	2.2 ± 1.0	2.0 ± 0.7	NS

Hospitalizations in past year (Median, IQR)	2.0 (1.3-4.8)	0.5 (0.0-3.25)	NS

Exacerbations in past year (Median, IQR)	6.0 (4.3-11.5)	3.0 (2.0-5.0)	P < 0.01

FEV_1_, l	1.8 ± 0.6	2.1 ± 1.0	NS

FEV_1_% predicted	65.0 ± 20.3%	73.4 ± 27.9%	NS

FVC, l	3.1 ± 1.0	3.4 ± 1.2	NS

FVC% predicted	92.3 ± 19.3%	95.4 ± 18.0%	NS

TLC % predicted	105.2 ± 13.9	111.9 ± 8.2	NS

RV % predicted	132.6 ± 32.4	148.9 ± 41.0	NS

RV/TLC %	43.6 ± 9.5	45.0 ± 10.3	NS

K_CO _% predicted	92.1 ± 14.6	92.4 ± 12.0	NS

Blood eosinophil count (10^9^/L)	0.3 ± 0.3	0.2 ± 0.2	NS

Serum IgE, IU/ml	118.0 (23.2-400.8)	149.5 (29.8-304.8)	**NS**

Serum IgG, g/L	9.3 ± 3.4	9.5 ± 2.7	NS

Serum IgA, g/L	2.3 ± 0.9	2.3 ± 0.8	NS

Serum IgM, g/L	1.1 ± 0.5	1.2 ± 0.5	NS

Data are presented as mean ± SEM or median (interquartile range)

Obesity was defined as a BMI of ≥ 30 kg/m^2^

*BDP *= Beclomethasone dipropionate; *BMI *= Body mass index; *BSA *= Body surface area; *FEV*_1 _= Forced expiratory volume in one second; *FVC *= Forced vital capacity; *TLC *= Total lung capacity; *RV *= Residual volume; *K_CO _*= Transfer coefficient to carbon monoxide

### Bacterial strains

**Figure 2:**
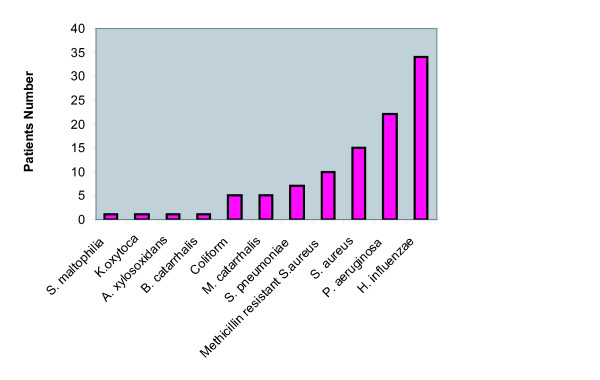
**Prevalence of bacterial strains detected in sputum by routine bacteriological culture in severe asthma patients**.

The bacterial strains detected in sputum from the 29 asthmatic patients included *H. influenzae, P. aeruginosa, S. aureus, methicillin-resistant S. aureus (MRSA), S. pneumoniae, M. catarrhalis, Coliform, B. catarrhalis, A. xylosoxidans, K.oxytoca *and *S. maltophilia *(Figure 2). *H. influenzae *was the most common pathogenic bacteria identified followed by *P. aeruginosa *and *S. aureus*. The majority had more than one bacterium cultured (16/29 patients; 55.2%) and 6/29 (20.7%) patients had more than two.

Twenty-six of the 56 patients had their sputum samples repeated once or more than once. These were performed particularly in those that had a positive bacterial growth. In 23 of the 29 bacteria-positive subjects, 14 had the same bacterial strains grown as in their first sputum culture samples, 2 had the different bacterial strains and 7 had no repeat positive bacterial cultures. In the 3 of the 27 bacteria-negative subjects, the culture remained negative.

### Airway wall thickness

**Figure 3:**
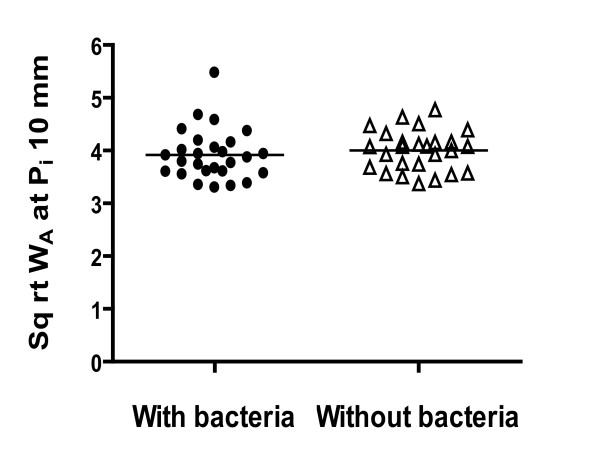
**Comparison airway dimensions of airways from severe asthma patients with or without bacteria cultured in sputum as assessed by √W_A _at P_i _of 10 mm**. There were no significant differences between the groups. The horizontal bars show the mean.

The HRCT scan measurements are presented in Table 2 and Figure 3. The radiologists did not report any gross evidence of bronchiectasis in these airways. The mean W_T_/D ratio and W_A_% were 21.3 ± 2.4 and 66.4 ± 5.4% in airways > 2 mm of asthmatics with positive bacterial cultures and 21.6 ± 2.7 and 67.5 ± 5.4% for those with asthmatics without bacteria, respectively. In airways ≤ 2 mm of asthma patients with bacteria, these measurements were 28.4 ± 4.2 and 80.2 ± 6.9%, while it was 27.3 ± 3.7 and 78.7 ± 6.6% in asthmatics without bacteria. There were no significant differences between the groups. In addition, √W_A _at P_i _of 10 mm was not different between the two groups (Figure 3).

**Table 2:** Airway measurements of HRCT scans in severe asthmatics with bacteria and without bacteria

	Asthma with positive bacteria	Asthma without positive bacteria*
	
	(n = 29)	(n = 27)
Total bronchi examined per group	401	349

*Large airways > 2 mm*		

Bronchi evaluated per patient	9.0 ± 3.7	9.2 ± 4.5

W_T_/D (%)	21.3 ± 2.4	21.6 ± 2.7

W_A _(%)	66.4 ± 5.4	67.5 ± 5.4

*Small airways ≤ 2 mm*		

Bronchi evaluated per patient	4.9 ± 3.3	3.9 ± 2.8

W_T_/D (%)	28.4 ± 4.2	27.3 ± 3.7

W_A _(%)	80.2 ± 6.9	78.7 ± 6.6

*All airways*		

√W_A _at P_i _of 10 mm	3.92 ± 0.09	4.00 ± 0.07

Data are presented as mean ± SEM

*D *= outer diameter; *P*_i _= airway internal perimeter; *W*_T _= wall thickness; *W*_A _= wall area; *W*_A_% = percentage wall area

*There were no significant differences between the groups for any of these parameters

**Figure 4:**
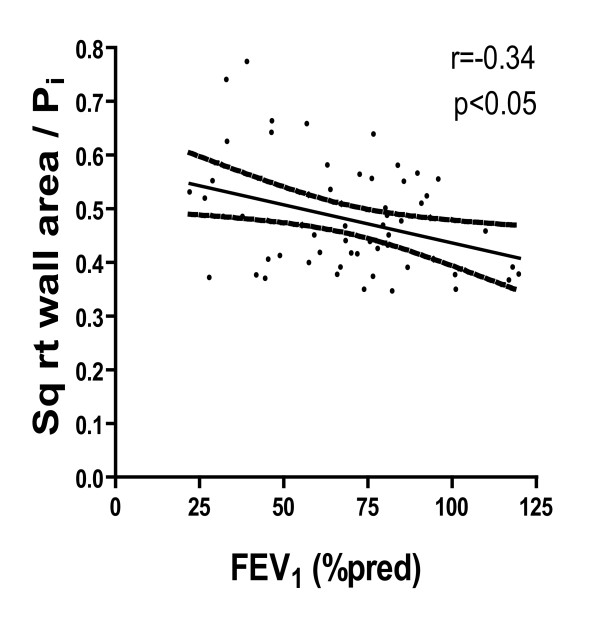
**Relationship between the ratio of √wall area to airway internal perimeter (P_i_) and FEV_1_% predicted in severe asthmatics for all the airways measured (r = -0.27, *p *< 0.05)**. The line of identity together with the 95% CI is shown.

When the 56 severe asthmatic subjects were analyzed together, √W_A _at P_i _of 10 mm showed no correlation with FEV_1_% predicted, while the ratio of √W_A_/P_i _was negatively correlated with FEV_1_% predicted (r = -0.34, *p *< 0.05, Figure 4).

### Factors associated with significant bacterial culture

Table 3 shows the cut-off values of variables for positive bacterial culture in sputum of severe asthmatic patients using logistic regression analysis. Univariate logistic analysis showed a significant association of the presence of bacteria on sputum culture with the duration of asthma and the number of exacerbations during the past year. Multivariate logistic analysis revealed both duration of asthma and the number of exacerbations in past year contribute to significant bacterial load in sputum, when odds ratio were adjusted for age, gender, smoking and FEV_1 _(% predicted).

**Table 3:** Univariate and multivariate analyses of predictive factors for positive bacterial load

*Univariate analysis*				
**Predicting factors**	**Odds ratio**		**95% CI**	**P-value**

Age	1.012		0.971-1.055	NS

Gender	1.111		0.362-3.413	NS

Duration of asthma	1.046		1.005-1.089	0.029

Exacerbations in past year	1.258		1.024-1.544	0.029

Smoking	1.764		0.602-5.171	NS

FEV_1 _(% pred)	0.983		0.962-1.005	NS

√W_A _at P_i _of 10 mm	0.627		0.175-2.248	NS

*Multivariate analysis*				

**Predicting factors**	**Odds ratio**	**Adjusted Odds** ratio*	**95% CI**	**P-value**

Duration of asthma	1.046	1.058	1.004-1.116	0.035

Exacerbations in past year	1.258	1.630	1.132-2.347	0.009

√W_A _at P_i _of 10 mm	0.627	0.631	0.168-2.372	NS

*Adjusted by age, gender, smoking and FEV_1 _(% predicted)

*CI *= confidence interval, *NS *= non-significant, *P*_i _= airway internal perimeter

## Discussion

We found a high prevalence of bacterial colonization by potentially pathogenic microorganisms in patients with chronic severe asthma who were in a relatively stable condition and who did not suffer from bronchiectasis. Using sputum culture for bacterial detection, we found that 52% showed positive sputum cultures with predominantly *H influenzae, P aeruginosa, S aureus and S pneumoniae strains*. These bacterial strains are similar to those previously reported in other airway conditions associated with bacterial colonization such as bronchiectasis and COPD [22,23], but higher than in a group of mild-to-moderate asthmatics or in asthmatics during an exacerbation [3,4]. There were no overt differences between the positive bacterial and negative bacterial growth patients in terms of most parameters related to severe asthma, apart from a significantly longer duration of asthma and greater number of exacerbations in the past year in patients with bacterial colonization. Logistic regression analysis showed that the longer duration of asthma and the number of exacerbations in the past year were significant risk factors for bacterial colonization. In a previous study of patients with severe asthma, we found that patients with severe asthma who had significant airflow obstruction reported a longer duration of asthma than those with normal lung function, together with greater evidence of airway wall thickening measured on HRCT scan [21].

Patients with severe asthma, as well as patients with moderate to severe COPD, are more vulnerable to exacerbations, which have been associated with respiratory pathogens, particularly viruses such as rhinovirus [24]. However, atypical bacterial infections such as Mycoplasma pneumonia have also been associated with exacerbations [25]. One study reported that 27% of patients with an exacerbation of asthma had evidence of bacterial growth in sputum [3]. However, since sputum of asthmatics who are not experiencing an exacerbation of their condition also show evidence of bacterial growth in a similar or even higher prevalence [4], one could question the relevance of these bacteria in the pathogenesis of these exacerbations. Interestingly, in many of the asthmatic patients who had a positive sputum culture also showed similar bacterial profile on repeat culture, indicating that these bacteria were most likely colonizing the lower airways.

In our univariate analysis, we found that a history of previous exacerbations to be a significant risk factor for positive bacterial cultures in sputum. The importance of these bacteria in the lower airways of asthmatic patients, particularly severe asthma, is currently unclear. This is likely related to a lack of information regarding the bacterial flora of the lower airways of patients with severe asthma and its variation with time or during worsening of asthma control. Two recent studies using a sensitive technique for determining the microbiome of the lower airways by 16S ribosomal RNA profiling have shown that there are bacterial species even in the lower airways of normal individuals and that this microbiome was significantly different in adult patients with asthma with evidence for pathogenic Proteobacteria, particularly Haemophilus spp [5,6].

An increase in airway wall thickness in asthma measured on HRCT scan has been related to the severity of asthma and to the degree of airflow obstruction [11,26-28]. A significant increase in airway wall thickness and size has been demonstrated in severe asthma compared to non-severe asthma and to non-asthmatic controls [9]. This study reports similar values of wall thickness expressed as W_T_/D ratio and W_A_% as those measured in our study. The increases in airway wall thickness measured on HRCT scans have been correlated with airway epithelial thickness measured on fixed tissue sections of airway biopsies, supporting the notion that these radiological abnormalities reflected airway wall remodeling processes [9]. On the other hand, the thickness of the reticular basement membrane has been shown to correlate with airway wall thickness measured radiologically in a mild-moderate group of asthma patients [29]. However, our studies reveal that the presence of bacteria in sputum is not associated with an increase in airway wall thickness as measured by two indices: W_T_/D and W_A_% at 2 airway sizes of or < 2 mm diameter. This lack of difference in airway wall thickness is unlikely to be influenced by studying different sizes of airways between the two groups since there was no difference in the √wall area (W_A_) at an airway internal perimeter (P_i_) of 10 mm, which represents a composite measure that takes into account the ranges of airway sizes measured for each subject [20]. Interestingly, we found a weak inverse correlation between the ratio of √wall area (W_A_) to P_i _with FEV_1_% predicted, but not between airway wall thickness or √wall area (W_A_) at an airway internal perimeter (P_i_) of 10 mm with FEV_1_% predicted, as previously reported [11,29].

Our data on the persistence of these bacteria in sputum shows that a high proportion of the positive sputum were also positive on repeat sputum testing with the same bacterial species, ie 14 out of 23 patients. However, in the other 9, there was either a different bacteria isolated or no bacteria was found. Further regular assessment is needed to determine whether these bacteria may be considered as colonizing the lower airways of patients with severe asthma. There may be various reasons why patients with severe asthma may be predisposed to greater bacterial colonization of their lower airways. Innate immune responses may be defective particularly in severe asthma. Toll-like receptor 4 gene expression is significantly decreased in airway neutrophils from asthmatics compared to healthy volunteers, together with the release of inflammatory cytokines [30]. Alveolar macrophages from patients with severe asthma compared to non-severe asthma were less able to phagocytose bacteria or apoptotic cells [31,32]. In addition, the Th2-polarised response may itself have a negative impact on the innate immune host response to bacterial infections [33,34]. The potential effect of chronic oral corticosteroid therapy, which up to 78% of our patients were taking, may suppress innate and acquired immune responses to bacterial infections [35]. The recent studies demonstrating an increased risk of asthma patients developing invasive pneumococcal disease or pneumonia [36,37] is likely to be a reflection of reduced innate immune responses in asthma, although the exact abnormality remains to be defined.

## Conclusions

Bacterial colonization of the lower airways is a common occurrence in patients with chronic stable severe asthma, but this was not related to the degree of airway wall thickness measured radiologically. We therefore conclude that bacterial colonization of asthmatic airways may not be the primary driver of airway wall remodeling, but it could be involved in other asthmatic processes.

## Abbreviations

A_L_: Luminal area; BMI: Body mass index; BSA: Body surface area; D: Diameter; FEV_1_: Forced expiratory volume in one second; FRC: Functional residual capacity; FVC: Forced vital capacity; HRCT: High resolution computed tomography; HU: Hounsfield Unit; K_CO_: Transfer coefficient to carbon monoxide; RV: Residual volume; TLC: Total lung capacity; W_A_: Wall area; W_T_: Wall thickness.

## Competing interests

AMG has been renumerated for participating on Advisory Board meetings organised by GSK, Novartis and Genentech, participated in Phase II and II studies with GSK and Novartis and has lectured for GSK, AZ and Novartis. KFC has received university grant monies from the Wellcome Trust, Medical Research Council, Asthma UK, NIH, and National Environmental Research Council (UK). He has also been renumerated for participating at Advisory Board meetings with GSK and Gilead, and for participating in speaking activities at the invitation of GSK and Novartis. Other authors have no declarations to make.

## Authors' contributions

QZ, CKH & AMG recruited the patients; QZ also complied the results and wrote the manuscript; RI, KD and DC analysed the HRCT scans; MS & KA analysed the sputum cultures; NSZ contributed to the idea and to the manuscript; KFC conceived the idea, directed the research and wrote the manuscript, and is the guarantor of the paper. All authors read and approved the final manuscript.
